# Phlorotannin Alleviates Liver Injury by Regulating Redox Balance, Apoptosis, and Ferroptosis of Broilers under Heat Stress

**DOI:** 10.3390/antiox13091048

**Published:** 2024-08-28

**Authors:** Zhong-Xiang Zhao, Yue-Ming Yuan, Zhi-Hui Zhao, Qing-Hua Yao, Xue-Qing Ye, Yao-Yao Wang, Hui-Mei Liu, Rajesh Jha, Balamuralikrishnan Balasubramanian, Wen-Chao Liu

**Affiliations:** 1 Department of Animal Science, College of Coastal Agricultural Science, Guangdong Ocean University, Zhanjiang 524088, China; zhaozhongxiang@stu.gdou.edu.cn (Z.-X.Z.); yuanyueming@stu.gdou.edu.cn (Y.-M.Y.); zhzhao@gdou.edu.cn (Z.-H.Z.); yaoqinghua1@stu.gdou.edu.cn (Q.-H.Y.); yexueqing1@stu.gdou.edu.cn (X.-Q.Y.); wangyaoyao@stu.gdou.edu.cn (Y.-Y.W.); liuhuimei@stu.gdou.edu.cn (H.-M.L.); 2 Department of Human Nutrition, Food and Animal Sciences, College of Tropical Agriculture and Human Resources, University of Hawaii at Manoa, Honolulu, HI 96822, USA; rjha@hawaii.edu; 3 Department of Food Science and Biotechnology, College of Life Science, Sejong University, Seoul 05006, Republic of Korea; bala.m.k@sejong.ac.kr

**Keywords:** broilers, heat stress, liver, oxidative stress, seaweed polyphenols

## Abstract

Heat stress (HS) poses a great challenge to the poultry industry by inducing oxidative damage to the liver, endangering the health and production of broilers. As an important type of seaweed polyphenols, phlorotannin has been shown to have antioxidant properties. The present study evaluated the protective effects of dietary phlorotannin on HS-induced liver injury in broilers based on oxidative damage parameters. A total of 108 twenty-one days old male Arbor Acres plus (AA+) broilers were randomly divided into three groups: TN group (thermoneutral, 24 ± 1 °C, fed with basal diet), HS group (HS, 33 ± 1 °C for 8 h/day, fed with basal diet), and HS + phlorotannin group (HS + 600 mg/kg phlorotannin). Each group has six replicate cages with six birds per cage. The feeding experiment lasted 21 days. At the termination of the feeding experiment (42 days old), samples were collected for analysis of morphological and biochemical features. The results showed that HS decreased the liver index, serum albumin (ALB) content, hepatic antioxidant enzymes activities of catalase (CAT), total superoxide dismutase (T-SOD), glutathione S-transferase (GST), and glutathione peroxidase (GSH-Px) (*p* < 0.05), while increasing the hepatic histopathology score, apoptosis rate, and malondialdehyde (MDA) content (*p* < 0.05) in 42-day-old broilers. Compared with the HS group, dietary phlorotannin improved the activities of antioxidant enzymes (GST and GSH-Px) but decreased the histopathology score and apoptosis rate in the liver (*p* < 0.05). Moreover, HS down-regulated hepatic mRNA expression of *CAT1*, *NQO1*, *HO-1*, and *SLC7A11* (*p* < 0.05), while up-regulated hepatic mRNA expression of *Keap1*, *MafG*, *IκBα*, *NF-κB P65*, *IFN-γ*, *TFR1*, *ACSL4*, *Bax,* and *Caspase-9* (*p* < 0.05). Compared with HS group, dietary phlorotannin up-regulated hepatic mRNA expression of *Nrf2*, *CAT1*, *MafF*, *GSTT1*, *NQO1*, *HO-1*, *GCLC*, *GPX1*, *TNF-α*, *Fpn1,* and *SLC7A11* (*p* < 0.05), while down-regulated hepatic mRNA expression of *IκBα*, *Bax*, *Caspase-9,* and *TFR1* (*p* < 0.05). In conclusion, dietary supplementation of 600 mg/kg phlorotannin could alleviate HS-induced liver injury via regulating oxidative status, apoptosis, and ferroptosis in broilers; these roles of phlorotannin might be associated with the regulation of the Nrf2 signaling pathway.

## 1. Introduction

With the continuous rise of global temperatures, heat stress (HS) has been a major challenge in restricting efficient poultry production worldwide [1]. Broilers are sensitive to HS because of their rapid growth, high metabolic rate, covered feathers, and limited sweat glands [2]. The decreased appetite is the typical characteristic of broilers suffering from HS, simultaneously experiencing physiological dysfunction and metabolic disorders, and the ultimate adverse consequence of HS is growth inhibition [3,4]. Therefore, HS has caused significant economic losses to the broiler industry [5]. HS not only impairs physiological functions and metabolic states but also induces oxidative damage to the organs of broilers [6,7]. Under HS conditions, broilers experience a sharp increase in respiratory rate and accelerated peripheral blood flow to expand heat dissipation [8,9]. As a result, reactive oxygen species (ROS) are produced in large quantities when broilers are exposed to HS; excessive ROS disrupts the redox balance, thereby leading to oxidative stress and adversely affecting organ function [10]. The detrimental effect of HS-induced oxidative stress on organ health is linked to pathological changes in histomorphology, apoptosis, and nucleic acid destruction [11,12]. Meanwhile, emerging research suggested that cellular ferroptosis is often accompanied by oxidative stress, which is a new mechanism of organ damage caused by HS [13,14]. Therefore, developing a sustainable strategy to prevent organs from oxidative attack is crucial for the health and production of broilers under HS.

As a central organ for nutrient metabolism and detoxification, the liver is essential for maintaining internal homeostasis and plays a major role in the rapid growth of broilers [15]. It has been confirmed that HS causes oxidative damage to the liver, specifically manifested in tissue lesions, suppressed antioxidant capacity, hepatocyte apoptosis, and ferroptosis, resulting in abnormal metabolism of broilers [16,17]. In the production practice of broilers, nutritional strategies have been implemented and proven to be viable in reducing the deleterious impacts of HS on liver function. Currently, phytochemicals are widely used in broiler nutrition to combat HS because of their multiple biological activities [18,19]. Polyphenols have been used as natural phytochemicals and antioxidants to alleviate HS-induced liver injury in broilers [20]. Available evidence suggests that the polyphenols, including curcumin and resveratrol, can act as the activators of nuclear factor erythroid 2-related factor 2 (Nrf2) and enhance the antioxidant performance via activating Nrf2-related antioxidant pathways, thus mitigating oxidative stress of liver in broilers challenged with HS [21,22]. Although previous studies have demonstrated the health benefits of polyphenols in heat-stressed broilers, the existing research mainly focused on the polyphenols from terrestrial plants; there are limited reports on marine-derived polyphenols.

Seaweed is one of the richest marine resources, containing many active biomolecules, and the seaweed extracts have attracted increasing attention from nutrition researchers in recent years because of their health promotion functions [23]. In addition, the enormous ocean area prompts seaweed products to be available commercially worldwide. Phlorotannin belongs to seaweed polyphenols and is extracted from marine brown algae, which contains several phenolic hydroxyl groups and has been found to possess various positive effects, such as free radical scavenging, antioxidant, and antibacterial properties in vitro [24,25]. Furthermore, phlorotannin has been revealed to reduce oxidative stress by up-regulating the Nrf2 signaling pathway based on model animals [26,27]. However, the hepatoprotective effect of phlorotannin and the underlying mechanisms in heat-stressed broilers are unclear. Therefore, this study aimed to explore the alleviating effect of dietary phlorotannin on HS-induced liver injury in broilers and reveal the potential molecular mechanisms.

## 2. Materials and Methods

### 2.1. Birds, Experimental Design, Management and Diet

A total of 180 one-day-old male Arbor Acres plus (AA+) broilers were obtained from the Zhanjiang Branch of Charoen Pokphand Group (Zhanjiang, Guangdong, China). The animal study was undertaken at the Poultry Experimental House of Guangdong Ocean University, and all experimental procedures were conducted with the approval of the Animal Care and Use Committee of Guangdong Ocean University (Zhanjiang, Guangdong, China). All broilers are fed in triple vertical cages in enclosed spaces with windows. The cages were made of iron, and each cage was 0.7 m long, 0.7 m wide, and 0.4 m high. The chicks were fed and watered using plastic discs with plastic feet at the bottom of the cage. As the broilers grew, we used large rectangular troughs for feeding and a round plastic hose for the watering system. All broilers are lighted with white fluorescent lamps. During the period from day 1 to day 20, all birds were reared indiscriminately under uniform conditions and fed a basal diet. At 21 days of age, 108 broilers with similar body weight (933.57 ± 5.87 g) and good health condition were selected and randomly divided into three groups: TN group (thermoneutral, 24 ± 1 °C, fed with basal diet), HS group (HS, 33 ± 1 °C for 8 h/day, 9:00 am to 17:00 pm, fed with basal diet), and HS + phlorotannin group (broilers under HS supplemented with 600 mg/kg phlorotannin). Phlorotannin is homogeneously mixed into broiler diets after graded premixing. There are six replicates in each group and six birds in each replicate. The feeding experiment lasted 21 days, and the relative humidity of all treatment groups in the chicken house was maintained at 65–75%. The supplemental level of phlorotannin was based on a previous study, which found that the inclusion of 400–600 mg/kg natural polyphenols in the basal diet mitigated oxidative damage to organs in broilers [28]. The products of phlorotannin with 98% purity were provided by Shaanxi Baichuan Biotechnology Co., Ltd. (Xi’an, Shaanxi, China). The basal diet formula is presented in Table 1, which was prepared based on the NRC (1994) and feeding standards of AA+ broilers. The basal diet was antibiotics-free, and the birds were allowed to drink and eat ad libitum throughout the experimental period.

### 2.2. Sample Collection

At the termination of the feeding experiment (42 days of age), 6 birds were randomly selected from each group (1 bird per replicate). Approximately 6 mL of blood was collected using an anticoagulant-free vacuum tube, and serum samples were obtained after the blood was allowed to coagulate and then centrifuged at 4000 rpm/min (15 min, 4 °C). Then, the serum samples were dispensed and stored at −80 °C. Then, the liver samples were collected and weighed after the euthanasia of birds. Liver samples are divided into different enzyme-free centrifuge tubes and stored in liquid nitrogen at −80 °C for further analysis.

### 2.3. Examination of Liver Histopathology and Liver Index

To examine liver histopathology and liver index, liver samples (n = 6) were photographed, weighed, and sampled. The liver tissues were washed with phosphate-buffered saline and stained with hematoxylin and eosin (H&E) after fixation with pre-prepared neutral buffered paraformaldehyde (4%). The liver histopathology was observed by HE-stained sections with an inverted fluorescence microscope (GD-30RFL) provided by Guangzhou Jidi Instrument Co., Ltd. (Guangzhou, Guangdong, China) under 200× and 400× magnification. Finally, the liver index and pathology score were calculated and assessed according to a previous study [29].

### 2.4. Assessment of Liver Function

The liver function parameters in the serum (n = 6) were analyzed using the commercial kits from Nanjing Jiancheng Bioengineering Institute (Nanjing, Jiangsu, China) and were in accordance with the instructions of the manufacturer, including the kits of albumin (ALB) (A028-1-1), alanine aminotransferase (ALT) (C009-2-1), and aspartate aminotransferase (AST) (C010-2-1). In addition, the total protein (TP) concentration of serum was analyzed using TaKaRa BCA Protein Assay Kit (T9300A) from Takara Biotechnology Co., Ltd. (Beijing, China).

### 2.5. Detection of Antioxidant Capability

For analyzing the antioxidant capacity, liver samples (n = 6) were homogenized with normal saline and collected the supernatant after centrifugation at 4000 rpm/min (5 min, 4 °C). The commercial kits of total antioxidant capacity (T-AOC) (A015-2-1), catalase (CAT) (A007-1-1), total superoxide dismutase (T-SOD) (A001-1-1), malondialdehyde (MDA) (A003-1-2), glutathione S-transferase (GST) (A004-1-1), and glutathione peroxidase (GSH-Px) (A005-1-2) were provided by Nanjing Jiancheng Bioengineering Institute (Nanjing, Jiangsu, China). The analysis of total protein concentration in liver samples using TaKaRa BCA Protein Assay Kit (T9300A) from Takara Biotechnology Co., Ltd. (Beijing, China). Specific assay operation methods were in accordance with the instructions of the kits.

### 2.6. Apoptosis Assay by TUNEL

The apoptotic cells in the liver were identified by terminal deoxynucleotidyl transferase-mediated dUTP nick end labeling (TUNEL) assay (n = 6). The slices were observed, and images were taken under a fluorescence microscope. Blue fluorescence is the normal cell nucleus, and positive apoptotic cells were marked as red. The numbers of apoptotic cells were quantified at a magnification of 200×. The rate of hepatocyte apoptosis was performed and calculated using ImageJ 1.51 (Bethesda, MD, USA).

### 2.7. Determination of mRNA Expression

Total RNA was extracted from liver samples (n = 6) using AG RNAex Pro Reagent (AG21101) provided by Accurate Biotechnology Co., Ltd. (Changsha, Hunan, China). The OD260/OD280 ratio was used to determine the purity of RNA, while agarose gel electrophoresis was used to detect the integrity of the extracted RNA. The cDNA was reverse transcribed using HIScript II Q RT SuperMix for qPCR (+gDNA wiper) kit (R223), which was obtained from Vazyme Co., Ltd. (Nanjing, Jiangsu, China). The cDNA was amplified by qRT-PCR in a CFX-96 real-time PCR detection system (BioRad, Irvine, CA, USA) and used the ChamQ Universal SYBR qPCR Master Mix kit (Q711), which was obtained from Vazyme Co., Ltd. (Nanjing, Jiangsu, China). The reaction system and conditions were designed strictly according to the kit’s instructions. In this experiment, β-actin was selected as the reference gene. The primer’s information is presented in Table 2. The 2^−∆∆Ct^ method was used for data processing, and the hepatocyte mRNA relative expression compared with the TN group was used to express the gene expression results.

### 2.8. Statistical Analysis

All data were analyzed using the one-way ANOVA procedure of SAS 9.4 (SAS, Cary, NC, USA). The significance of differences between groups was analyzed using Tukey’s multiple comparisons. The correlation analysis was generated in RStudio version 2023.06.2+561 using the statistical software R (R version R-4.3.0) and library (corrplot) version 0.92, and the correlation coefficient analysis was performed with Spearman. *p* < 0.05 was considered to be statistically significant, and 0.05 ≤ *p* < 0.10 was considered to be a tendency.

## 3. Results

### 3.1. Liver Histopathology

**Figure 1:**
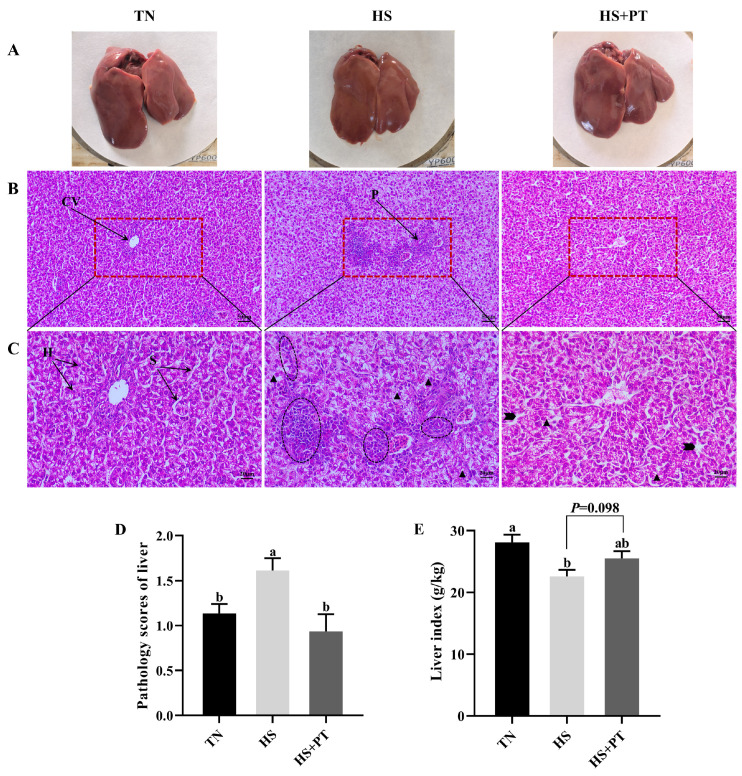
Effect of Phlorotannin on liver changes in heat-stressed broilers at 42 days old (n = 6). (**A**) Observation of the liver by the naked eye. Histopathological changes in the liver were stained by hematoxylin and eosin ((**B**) ×200, (**C**) ×400). (**D**) Pathology scores of broiler liver. (**E**) Effects of phlorotannin on the liver index of heat-stressed broilers. TN: thermoneutral group, the broilers reared at 24 ± 1 °C throughout the experimental period, fed with basic diet; HS: heat stress group, the broilers reared at 33 ± 1 °C for 8 h/day (9:00 am to 17:00 pm), fed with basic diet; HS + PT: HS + phlorotannin group, the broilers reared at 33 ± 1 °C for 8 h/day (9:00 am to 17:00 pm), fed with 600 mg/kg phlorotannin in the basic diet. CV: central vein; H: hepatocyte; S: hepatic sinusoid; P: portal area. Inflammatory cell infiltration (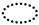), vacuolization (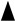), dilated hepatic sinusoids (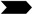). ^ab^ Indicates that the same columns carrying various superscripts are significantly different at *p* < 0.05.

Figure 1A showed the superficial morphology of the liver between the three groups; all liver samples had a smooth, reddish-brown surface and were soft and flexible, with clearly visible edges. No superficial pathological changes, such as necrosis and hemorrhage, were found in the liver. Figure 1B (200×) and Figure 1C (400×) indicated the histopathological changes in liver tissues. Compared with the TN group, HS caused vacuolization of hepatocytes, disordered hepatocyte arrangement, the loss of hepatic cords and sinusoids, and the infiltration of neutrophils and lymphocytes. Compared with the HS group, dietary phlorotannin reduced the infiltration of neutrophils and lymphocytes, restored the normal cellular architecture of liver tissues with pronounced hepatic cords and sinusoids, and resulted in the orderly arrangement of hepatocytes. As presented in Figure 1D, broilers in the HS group had a higher histopathological score of the liver than those in the TN group (*p* < 0.05). In contrast, dietary phlorotannin decreased the histopathological score of the liver in broilers under HS (*p* < 0.05). As illustrated in Figure 1E, the liver index of broilers in the HS group was lower than those in the TN group (*p* < 0.05). Dietary phlorotannin had a trend to improve the liver index of broilers under HS (*p* = 0.098).

### 3.2. Liver Function

Liver function-related indexes in the serum are shown in Table 3. Broilers in the HS group had a lower serum ALB content than those in the TN group (*p* < 0.05). Dietary phlorotannin supplementation had a trend to increase serum TP levels in broilers exposed to HS (*p* = 0.098).

### 3.3. Antioxidant Capacity

As presented in Table 4, compared with the TN group, HS increased the hepatic MDA content of broilers (*p* = 0.029) while reducing the hepatic activities of CAT, T-SOD, GST, and GSH-Px (*p* < 0.05). Compared with the HS group, dietary phlorotannin supplementation enhanced the hepatic activities of GST and GSH-Px in broilers at 42 days old (*p* = 0.022, *p* = 0.030, respectively). Meanwhile, compared with the TN group, dietary phlorotannin supplementation in heat-stressed broilers enhanced the hepatic MDA content (*p* = 0.008) while reducing the hepatic activities of CAT, GST, and GSH-Px (*p* < 0.05).

### 3.4. Hepatocyte Apoptosis

**Figure 2:**
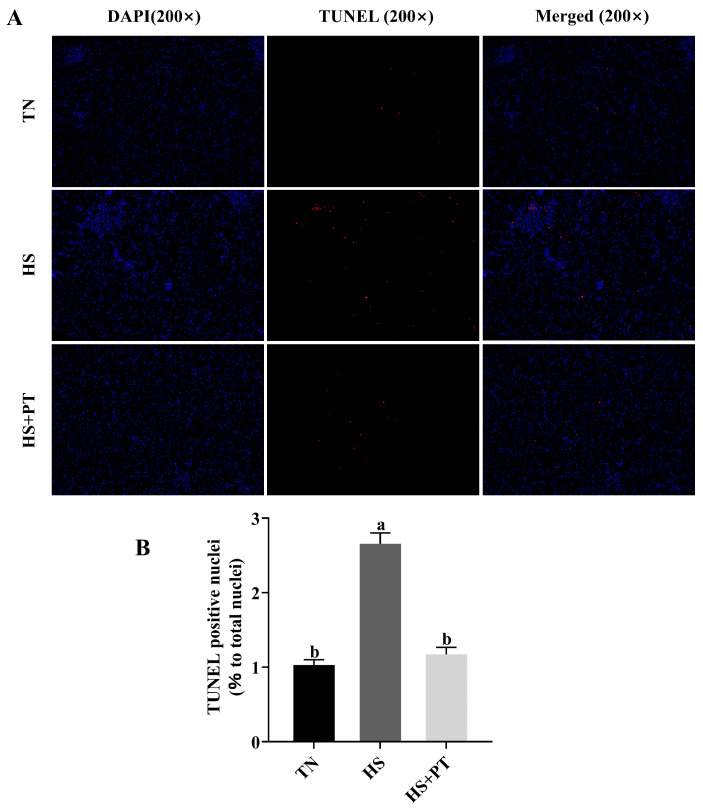
Effects of phlorotannin on apoptosis in the liver of heat-stressed broilers (TUNEL assay). (**A**) TUNEL staining results of broiler liver tissues (200×), blue fluorescence is a normal cell nucleus, and apoptotic cells were dyed red. (**B**) Percentage of TUNEL-positive cells. The results are represented as mean ± SEM (n = 6). TN: thermoneutral group, the broilers reared at 24 ± 1 °C throughout the experimental period, fed with basic diet; HS: heat stress group, the broilers reared at 33 ± 1 °C for 8 h/day (9:00 am to 17:00 pm), fed with basic diet; HS + PT: HS + phlorotannin group, the broilers reared at 33 ± 1 °C for 8 h/day (9:00 am to 17:00 pm), fed with 600 mg/kg phlorotannin in the basic diet. DAPI: 4’,6-diamidino-2-phenylindole, a fluorescent dye that can bind strongly to DNA; TUNEL: terminal dexynucleotidyl transferase (TdT)-mediated dUTP nick end labeling; Merged: a double staining technique; ^ab^ Indicates that the same columns carrying various superscripts are significantly different at *p* < 0.05.

As shown in Figure 2, broilers in the HS group had a higher apoptosis rate of hepatocytes compared with the broilers in the TN group (*p* < 0.05), and phlorotannin intervention reduced the apoptosis rate of hepatocytes in broilers challenged with HS (*p* < 0.05).

### 3.5. Determination of Antioxidant-Related Gene Expression

**Figure 3:**
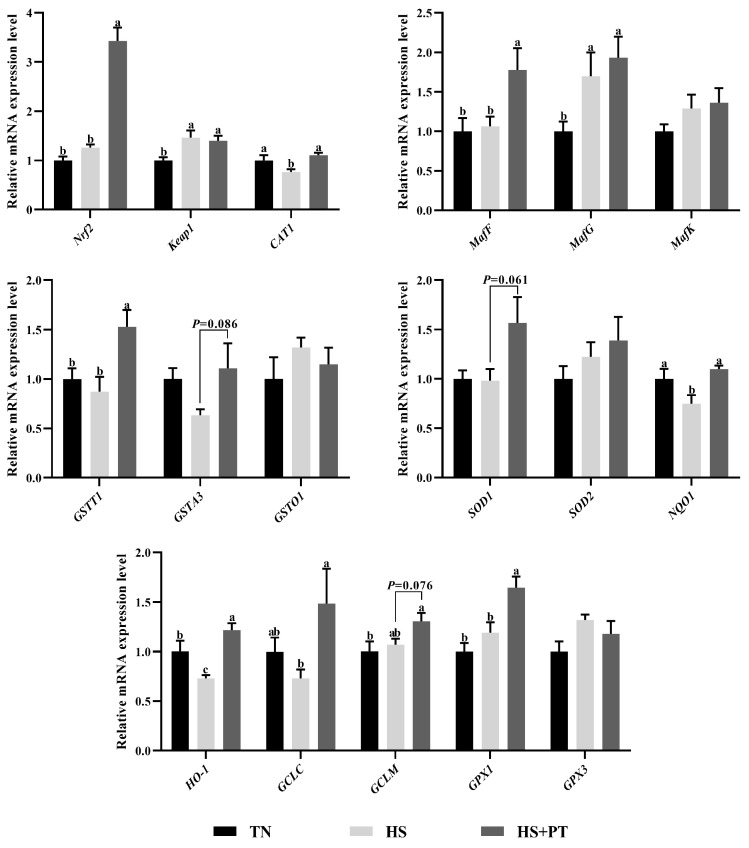
Effect of dietary phlorotannin on mRNA expression of genes related to antioxidants. TN: thermoneutral group, the broilers reared at 24 ± 1 °C throughout the experimental period, fed with basic diet; HS: heat stress group, the broilers reared at 33 ± 1 °C for 8 h/day (9:00 am to 17:00 pm), fed with basic diet; HS + PT: HS + phlorotannin group, the broilers reared at 33 ± 1 °C for 8 h/day (9:00 am to 17:00 pm), fed with 600mg/kg phlorotannin in the basic diet. *Nrf2*: nuclear factor-erythroid factor 2-related factor 2; *Keap1*: kelch-like associated protein 1; *CAT1*: y+ cationic amino acid transporter 1; *MafF*: MAF bZIP transcription factor F; *MafG*: MAF bZIP transcription factor G; *MafK*: MAF bZIP transcription factor K; *GSTT1*: glutathione S-transferase theta 1; *GSTA3*: glutathione S-transferase alpha 3; *GSTO1*: glutathione S-transferase omega 1; *SOD1*: superoxide dismutase 1; *SOD2*: superoxide dismutase 2; *NQO1*: NAD(P)H dehydrogenase, quinone 1; *HO-1*: heme oxygenase 1; *GCLC*: glutamate-cysteine ligase catalytic subunit; *GCLM*: glutamate-cysteine ligase modifier subunit; *GPX1*: glutathione peroxidase 1; *GPX3*: glutathione peroxidase 3. The results are represented as mean ± SEM (n = 6). ^abc^ Indicates that the same columns carrying various superscripts are significantly different at *p* < 0.05.

As illustrated in Figure 3, compared with the TN group, HS exposure decreased the hepatic mRNA expression level of *CAT1*, *NQO1,* and *HO-1* (*p* < 0.05) and increased the hepatic mRNA expression level of *Keap1* and *MafG* (*p* < 0.05). In contrast, phlorotannin supplementation increased the hepatic mRNA expression level of *Nrf2*, *CAT1*, *MafF*, *GSTT1*, *NQO1*, *HO-1*, *GCLC,* and *GPX1* (*p* < 0.05) and had a trend to increase the hepatic mRNA expression level of *GSTA3*, *SOD1,* and *GCLM* in broilers under HS (*p* = 0.086, *p* = 0.061, *p* = 0.076, respectively).

### 3.6. Determination of Inflammation-Related Gene Expression

**Figure 4:**
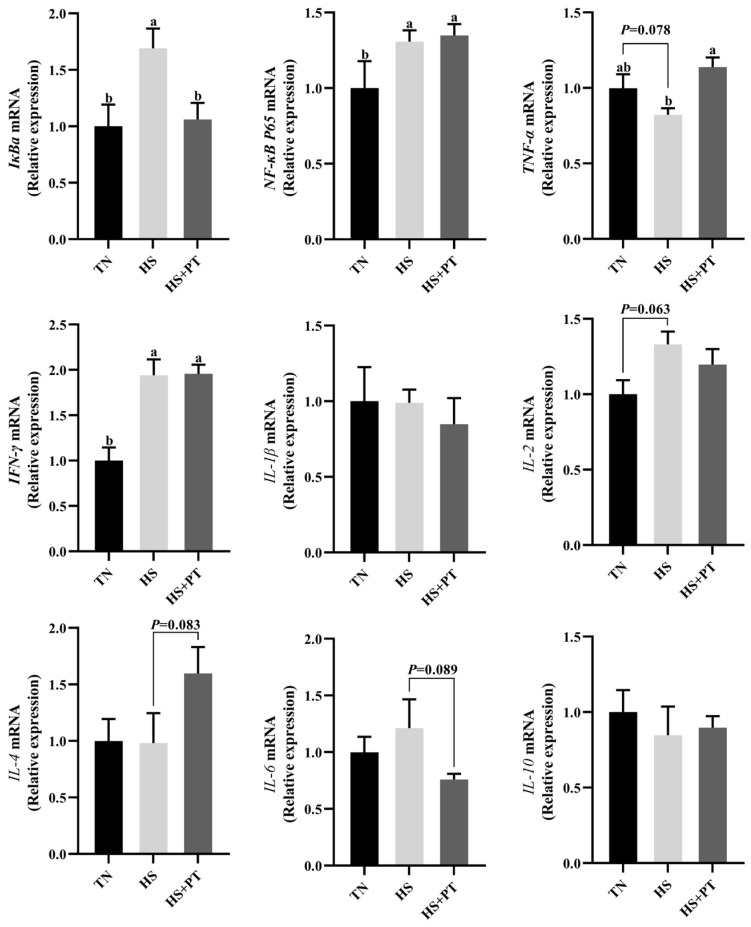
Effect of dietary phlorotannin on mRNA expression levels of inflammation-related genes. TN: thermoneutral group, the broilers reared at 24 ± 1 °C throughout the experimental period, fed with basic diet; HS: heat stress group, the broilers reared at 33 ± 1 °C for 8 h/day (9:00 am to 17:00 pm), fed with basic diet; HS + PT: HS + phlorotannin group, the broilers reared at 33 ± 1 °C for 8 h/day (9:00 am to 17:00 pm), fed with 600mg/kg phlorotannin in the basic diet. *IκBa*: *NF-κB* inhibitor alpha; *NF-κB P65*: nuclear factor κappa B P65; *TNF-α*: tumor necrosis factor-α; *IFN-γ*: interferon gamma; *IL-1β*: interleukin 1 beta; *IL-2*: interleukin 2; *IL-4*: interleukin 4; *IL-6*: interleukin 6; *IL-10*: interleukin 10. The results are represented as mean ± SEM (n = 6). ^ab^ Indicates that the same columns carrying various superscripts are significantly different at *p* < 0.05.

As displayed in Figure 4, HS increased the mRNA expression level of *IκBa*, *NF-κB P65,* and *IFN-γ* (*p* < 0.05) in the liver compared with the TN group. The HS exposure caused an increased tendency of *IL-2* (*p* = 0.063) and a decreased tendency of *TNF-α* (*p* = 0.078) on the mRNA expression levels in the liver compared with the TN group. Dietary phlorotannin increased the mRNA expression level of *TNF-α* (*p* < 0.05) but decreased the mRNA expression level of *IκBa* (*p* < 0.05) in the liver of heat-stressed broilers. In addition, dietary phlorotannin tended to reduce the mRNA expression level of *IL-6* (*p* = 0.089) and tended to increase the mRNA expression level of *IL-4* (*p* = 0.083) in the liver of heat-stressed broilers. Worthy of attention, the HS+PT group, similar to the HS group, showed elevated mRNA expression levels of *NF-κB P65* and *IFN-γ* (*p* < 0.05) in the liver compared with the TN group.

### 3.7. Determination of Ferroptosis and Apoptosis-Related Gene Expression

**Figure 5:**
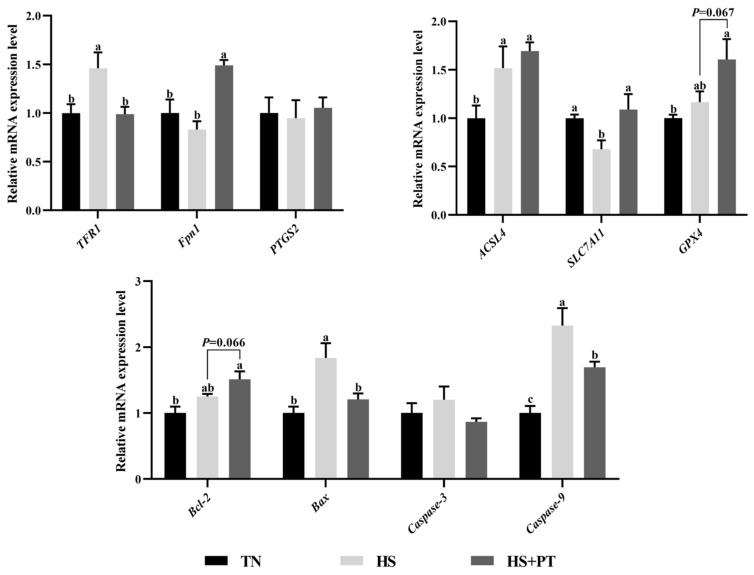
Effect of dietary phlorotannin on mRNA expression levels of ferroptosis and apoptosis regulatory genes. TN: thermoneutral group, the broilers reared at 24 ± 1 °C throughout the experimental period, fed with basic diet; HS: heat stress group, the broilers reared at 33 ± 1 °C for 8 h/day (9:00 am to 17:00 pm), fed with basic diet; HS + PT: HS + phlorotannin group, the broilers reared at 33 ± 1 °C for 8 h/day (9:00 am to 17:00 pm), fed with 600 mg/kg phlorotannin in the basic diet. *TFR1*: transferrin receptor; *Fpn1*: solute carrier family 40 member 1; *FTH1*: ferritin heavy chain 1; *ACSL4*: acyl-CoA synthetase long chain family member 4; *SLC7A11*: solute carrier family 7 member 11; *GPX4*: glutathione peroxidase 4; *PTGS2*: prostaglandin-endoperoxide synthase 2; *Bcl-2*: B cell leukemia/lymphoma 2; *Bax*: BCL2 associated X; *Caspase-3*: cysteinyl aspartate specific proteinase 3; *Caspase-9*: cysteinyl aspartate specific proteinase 9. The results are represented as mean ± SEM (n = 6). ^abc^ Indicates that the same columns carrying various superscripts are significantly different at *p* < 0.05.

As presented in Figure 5, compared with the TN group, HS down-regulated the hepatic mRNA expressions level of *SLC7A11* (*p* < 0.05) and up-regulated the level of *TFR1*, *ACSL4*, *Bax,* and *Caspase9* (*p* < 0.05). Phlorotannin supplementation up-regulated the hepatic mRNA expression level of *Fpn1* and *SLC7A11* (*p* < 0.05) and down-regulated the hepatic mRNA expression level of *TFR1*, *Bax,* and *Caspase9* compared with broilers under HS (*p* < 0.05). In addition, dietary phlorotannin tended to increase the mRNA expression level of *GPX4* and *Bcl-2* in the liver of heat-stressed broilers (*p* = 0.067, *p* = 0.066, respectively).

### 3.8. Correlation Analysis

**Figure 6:**
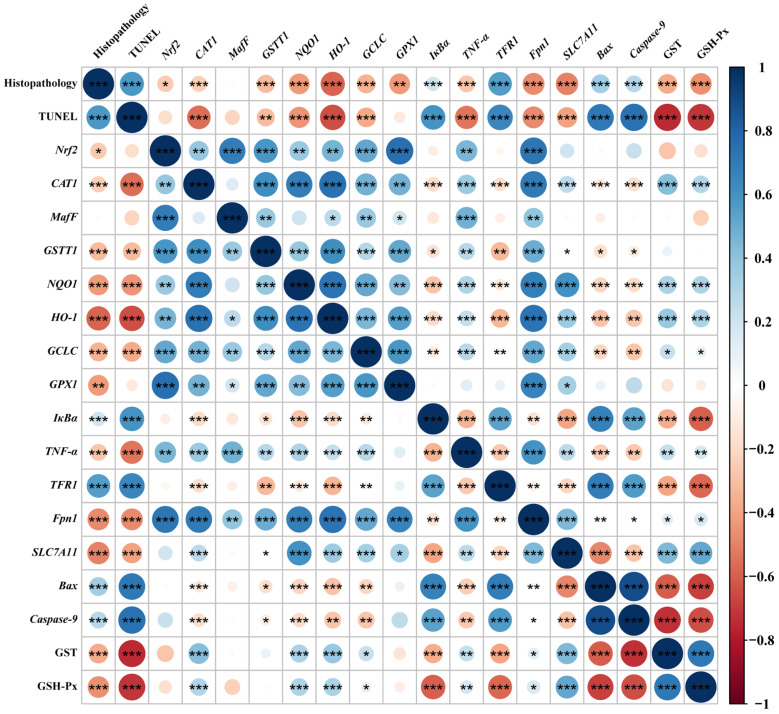
Spearman correlation analysis. The color legend on the right indicates correlation coefficient values by color. The value corresponding to the intermediate heat map is Spearman correlation coefficient r, which is between −1 and +1. When r < 0, it is a negative correlation, and when r > 0, it is a positive correlation. Histopathology: the pathological score of broiler liver; TUNEL: the apoptosis rate of broiler liver; *Nrf2*: nuclear factor-erythroid factor 2-related factor 2; *CAT1*: y+ cationic amino acid transporter 1; *MafF*: MAF bZIP transcription factor F; *GSTT1*: glutathione S-transferase theta 1; *NQO1*: NAD(P)H dehydrogenase, quinone 1; *HO-1*: heme oxygenase 1; *GCLC*: glutamate-cysteine ligase catalytic subunit; *GPX1*: glutathione peroxidase 1; *IκBa*: *NF-κB* inhibitor alpha; *TNF-α*: tumor necrosis factor-α; *TFR1*: transferrin receptor; *Fpn1*: solute carrier family 40 member 1; *SLC7A11*: solute carrier family 7 member 11; *Bax*: BCL2 associated X; *Caspase-9*: cysteinyl aspartate specific proteinase 9; GST, glutathione S-transferase; GSH-Px, glutathione peroxidase. * *p* < 0.05, ** *p* < 0.01, *** *p* < 0.001.

Spearman’s correlation analysis was performed with selected indicators that were significantly differentially affected by dietary phlorotannin (Figure 6). The relative mRNA expression level of *Nrf2* in the liver showed a positive correlation with the relative mRNA expression level of *MafF*, *GSTT1*, *GCLC*, *GPX1*, *Fpn1*, *CAT1*, *NQO1*, *HO-1,* and *TNF-α* (*p* < 0.01). In addition, the relative mRNA expression level of *Nrf2* showed a negative correlation with pathological score (*p* < 0.05). The relative mRNA expression level of *TFR1* showed a positive correlation with the histopathological score and the apoptosis rate (*p* < 0.001) and had a negative correlation with the expression of antioxidant-related genes (*p* < 0.01). The relative mRNA expression level of *Fpn1* and *SLC7A11* indicated a negative correlation with the histopathological score and the apoptosis rate (*p* < 0.001). In addition, the relative mRNA expression level of *Bax* and *Caspase-9* showed a positive correlation with the histopathological score and the apoptosis rate (*p* < 0.001), while it had a negative correlation with the expression of antioxidant-related genes (*p* < 0.05).

## 4. Discussion

The liver plays a major role in nutrient metabolism and health maintenance of broilers. However, the liver is the main target organ for HS attacks, and HS causes liver injury and disrupts internal homeostasis, thereby reducing the production performance of broilers [30]. It has been confirmed that broilers under HS had a higher level of AST in the serum, and there were abnormal liver indices and pathological changes in the liver [16,31]. Similarly, the present study found that HS decreased the serum ALB content and liver index, whereas it increased the pathological score of the liver. ALB is a protein synthesized by the liver, and the lower serum ALB level and liver index indicate HS-induced liver injury. Additionally, the histological changes suggested that there was hepatic inflammatory infiltration of broilers exposed to HS. In this study, dietary phlorotannin reversed (or had a trend) the deleterious influence of HS on liver function indicators of blood, liver index, and hepatic histomorphology. In accordance with our results, previous reports showed that phlorotannin exerted hepatoprotective activity in hepatocytes (HepG2 cells) [32,33,34]. Kang et al. [35] also demonstrated that dietary phlorotannin mitigated ethanol-induced liver damage by restoring the serum liver function parameters and the hepatic morphology in mice. Since we are the first to disclose the preventive effect of phlorotannin on liver injury in heat-stressed broilers, there are no direct studies for comparison. Research on other plant-derived polyphenolic substances suggested that curcumin and resveratrol could improve the liver function and lymphocyte infiltration of broilers challenged with HS [21,36]; these findings are similar to our results. Therefore, according to the current experiment, supplying 600 mg/kg phlorotannin alleviated HS-induced liver injury of broilers.

When broilers are exposed to HS conditions, excessive ROS is produced, and the redox balance is disrupted, thus causing oxidative damage to organs [6]. Oxidative stability depends on the balance between antioxidant enzymes and peroxides; it has been proved that HS results in liver oxidative stress by inhibiting antioxidant enzyme activity and increasing MDA content [3,30]. Consistent with previous studies, we found that broilers under HS had lower antioxidant enzyme activity, including CAT, T-SOD, GST, and GSH-Px, while they had a higher MDA level in the liver. Polyphenols are natural antioxidants and have shown great potential in maintaining redox balance in heat-stressed broilers. For instance, dietary inclusion of 100–200 mg/kg curcumin enhanced the hepatic GSH-Px and GST activity in broilers under HS [37]. Supplementation of 400 mg/kg resveratrol increased the activity of SOD and GSH-Px but reduced the MDA content in the liver in broilers during HS [21]. Similarly, the present study observed that the addition of 600 mg/kg phlorotannin in the heat-stressed broiler’s diet improved the GST and GSH-Px activity of the liver. However, dietary supplementation with phlorotannin did not increase CAT and T-SOD activity in heat-stressed broilers. The exact reason needs to be further investigated, and we speculate that it may be related to the different antioxidant mechanisms of various types of polyphenols. It is worth noting that although we found that GST and GSH-Px activity were elevated by phlorotannin, the clearance of MDA does not seem to be ideal. Perhaps due to the poor effect of phlorotannin on enhancing T-SOD activity, resulting in limited clearance of MDA, because SOD can act as an efficient MDA-eliminating enzyme [38]. In addition, there are various free radicals and peroxides in the body, but we only detected MDA; phlorotannin may have a clearing effect on other peroxides, so it is necessary to detect other free radicals and peroxides in future studies, such as ROS and LPO. Nrf2/Keap1 is a key signaling pathway that regulates antioxidant performance. Under normal physiological conditions, Nrf2 and Keap1 combine to form dimers in the cytoplasm; the activation of Nrf2 can dissociate from Keap1 and undergo nuclear translocation, which then binds to the promoter region of antioxidant genes to promote their transcription [38]. In this study, dietary phlorotannin up-regulated the hepatic *Nrf2* expression and the expression of antioxidant genes, including *CAT1*, *GSTT1*, *NQO1*, *HO-1*, *GCLC,* and *GPX1* in broilers challenged with HS. This indicates that phlorotannin may attenuate HS-induced oxidative damage to the liver by activating the Nrf2/Keap1 pathway in broilers. Accordantly, earlier studies have reported that dietary phlorotannin could activate the Nrf2 pathway and alleviate oxidative stress in model animals [26,27]. In addition, it has been demonstrated that multiple polyphenols acted as activators of Nrf2 and were associated with enhanced antioxidant capacity. For instance, natural polyphenols (quercetin) have been reported to improve antioxidant performance by regulating Nrf2 signal transduction, thereby antagonizing oxidative stress in broilers [39]. However, due to the scarcity of studies on phlorotannin in poultry, the current study only provides preliminary clues on the mechanism of action; further research on the specific molecular mechanisms underlying the regulation of the Nrf2/Keap1 pathway by phlorotannin is necessary.

The adverse consequences of HS on broilers are not limited to oxidative stress but also include inflammatory damage to organs. HS disrupts the balance between the secretion of anti-inflammatory and pro-inflammatory cytokines, leading to an inflammatory response in broilers [9]. Previously, Liu et al. [31] reported that chronic HS promoted liver inflammation by enhancing the NF-κB signaling pathway, thus reducing liver function and health in broilers. In the present study, HS resulted in up-regulation of *IκBα*, *NF-κB P65,* and *IFN-γ* expression, suggesting that HS altered the expression of inflammatory cytokines and affected immune-related signaling pathway in the liver. Nevertheless, dietary phlorotannin only showed the ability to reduce *IκBα* expression but increased the *TNF-α* expression level of the liver in heat-stressed broilers. In fact, in addition to the excellent antioxidant properties, natural polyphenols also exhibit anti-inflammatory activity in broilers. He et al. [40] demonstrated that dietary resveratrol could modulate inflammatory cytokines mRNA expression in the spleen of broilers under HS, mainly manifested in down-regulating *NF-κB P65*, *IL-1β*, *IL-4,* and *IL-6* expression, up-regulating *IFN-γ* expression level. He et al. [41] found that resveratrol supplementation decreased serum concentration of IL-1β, IL-4, and IL-6 in broilers exposed to HS. Our results are inconsistent with previous research, and the phlorotannin had a weak effect on liver inflammation of heat-stressed broilers. The possible reason is that there are differences in the chemical structure of multiple polyphenols, and the activity of immune regulation also varies. Again, it may also be due to the limited clearance effect of phlorotannin on MDA in this study, which resulted in the observation of partial pro-inflammatory and weak anti-inflammatory effects, as MDA has been shown to induce the release of pro-inflammatory factors [9]. Therefore, the inflammatory regulatory role of polyphenols may be related to antioxidant activity [42], but the anti-inflammatory ability of different polyphenols still needs further confirmation.

It has been validated that oxidative stress-induced cell death in broilers under HS is the main cause of organ dysfunction [10,12]. Apoptosis is a common form of cell death; the Bcl-2 family is an important regulatory factor mediating the endogenous pathway of apoptosis, Bcl-2 is an anti-apoptotic protein, while Bax is a typical pro-apoptotic protein, and the Caspase-3 and Caspase-9 have also been reported to be associated with promoting apoptosis [43]. Previous reports showed that HS increased apoptosis rate and up-regulated the mRNA expression of *Caspase-3* but down-regulated the mRNA expression of *Bcl-2* in the liver of broilers [16]. Consistently, the present study found that heat-stressed broilers had a higher hepatic apoptosis rate and mRNA expression level of *Bax* and *Caspase-9*. It is worth noting that dietary phlorotannin reduced hepatocyte apoptosis and decreased hepatic mRNA expression of *Bax* and *Caspase-9* in broilers under HS. Similar results were obtained by Zhang et al. [44], who demonstrated that resveratrol mitigated HS-induced splenic apoptosis, and this beneficial effect of resveratrol is attributable to the modulation of the redox status in heat-stressed broilers. There is evidence that the Nrf2 pathway interacts with apoptosis. The activation of Nrf2 signaling and the enhancement of antioxidant capacity can inhibit the generation of pro-apoptotic proteins, thereby reducing apoptosis [45]. In addition, we found that the apoptosis-related parameters were negatively correlated with the Nrf2-mediated antioxidant molecule’s expression. Suggesting that HS elevated hepatocyte apoptosis through oxidative stress in broilers, and the anti-apoptotic role of phlorotannin is most likely due to its antioxidant function and the regulation of the Nrf2 pathway.

In addition to apoptosis, whether HS causes other forms of cell death, such as ferroptosis, has not been widely investigated. Emerging research suggests that HS leads to ferroptosis in the liver and muscle cells of broilers [13,14,17]. Similarly, in this study, HS negatively affected the mRNA expression of ferroptosis-related molecules, including increased *TFR1* expression and decreased *Fpn1* and *SLC7A11* expression, and phlorotannin could reverse these adverse changes induced by HS. Recently, the anti-ferroptosis effects of natural polyphenols have been reported in model animals and human cells, such as curcumin [46,47], resveratrol [48,49], and quercetin [50,51]. As a new type of cell death, ferroptosis has become a current research hotspot, and it has been confirmed that ferroptosis is a new way of oxidative damage [52]. Ferroptosis is iron-dependent, and various stressors result in abnormal iron metabolism and transport, causing intracellular Fe^2+^ overload and ferroptosis. TFR1 and Fpn1 are transferrin proteins with different functions. TFR1 is mainly responsible for transporting Fe^2+^ into cells, while Fpn1 mainly mediates Fe^2+^ transfer to cells. SLC7A11 is a transmembrane transport protein that promotes the transport of cysteine within cells and the synthesis of glutathione (GSH), and the GSH is a key molecule in defense against ferroptosis, so SLC7A11 is usually regarded as an anti-ferroptosis factor [53]. Simultaneously, in the current study, correlation analysis showed that the expression of *TFR1* was negatively linked with liver histopathology, and the expression of *Fpn1* and *SLC7A11* was positively linked with liver histopathology. Therefore, phlorotannin may alleviate HS-induced ferroptosis of hepatocytes by regulating the expression of transferrin and anti-ferroptosis molecules. Interestingly, the Nrf2 pathway has been found to regulate the expression of transferrin and SLC7A11 [54], and the association analysis of this study also verified this conclusion. However, although this study reveals for the first time the potential of phlorotannin to reduce HS-induced hepatocyte ferroptosis in broilers, further research is essential to explore the relevant mechanisms of action.

## 5. Conclusions

**Figure 7:**
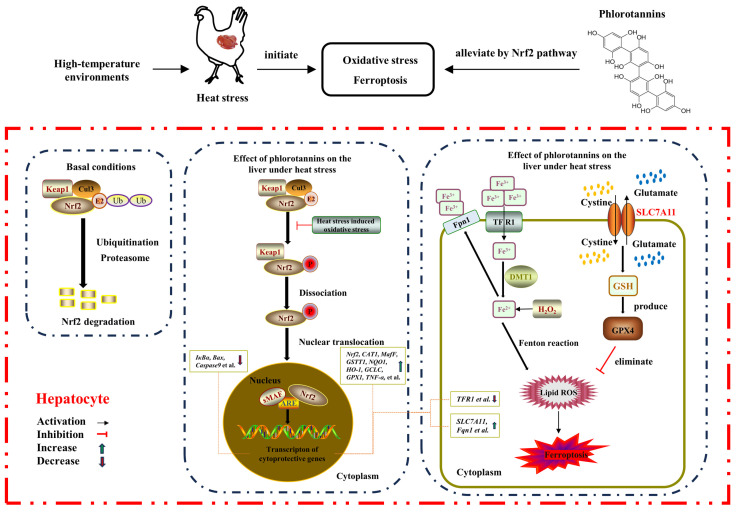
The proposed mechanism of phlorotannin alleviates heat stress-induced liver injury by regulating redox, apoptotic, and ferroptosis signaling pathways in broilers.

On the whole, HS resulted in liver injury by disrupting serum liver function indicators, increasing hepatic histopathology, MDA level, and apoptosis rate, and reducing the antioxidant enzyme activity of broilers. Dietary supplementation with 600 mg/kg phlorotannin could attenuate HS-induced liver injury by regulating redox homeostasis, apoptosis, and ferroptosis in broilers; this response may be related to the Nrf2 signaling pathway (Figure 7). This study provides novel insights into the hepatoprotective role of phlorotannin in heat-stressed broilers, promoting the development of anti-HS feed additives in broiler production. There are still some shortcomings in this study, such as the absence of a protein level for validation of the molecular mechanism of phlorotannin in heat-stressed broilers. In the next step, further research is needed to explore the relevant mechanisms of phlorotannin.

## Figures and Tables

**Table 1:** Composition and nutrient levels of the basal diet (air-dry basis).

Item	Contents (%)
Ingredients	
Corn	55.00
Soybean meal	34.82
Wheat bran	2.00
Soybean oil	5.00
Limestone	0.50
CaHPO_4_	1.60
Salt	0.30
*DL*-Met	0.18
*L*-Lysine	0.10
Choline chloride	0.10
Vitamin premix ^1^	0.20
Mineral premix ^2^	0.20
Total	100.00
Nutrient levels	
Metabolizable energy ^3^	12.82
Crude protein	19.92
Ca	0.93
Available phosphorus	0.44
Methionine	0.45
Lysine	1.30
Methionine + cystine	0.72

^1^ The vitamin premix provides the following per kg diet: vitamin A 9000 IU, vitamin D3 3240 IU, vitamin E 6 IU, vitamin K3 0.75 mg, vitamin B1 1.5 mg, vitamin B2 4.5 mg, vitamin B6 1.5 mg, niacinamide 10.5 mg, folic acid 0.45 mg, pantothenic acid 9 mg; ^2^ The mineral premix provides following per kg diet: Cu (CuSO_4_ · 5H_2_O) 5~10 mg, Fe (FeSO_4_) 45~120 mg, Mn (MnSO_4_) 45~85 mg, Zn (ZnSO_4_) 50~80 mg, Se (Na_2_SeO_3_) 0.2 mg, I (KI) 0.15 mg; ^3^ Metabolizable energy is calculated values, while the others were measured values. The unit of metabolizable energy is kcal/kg.

**Table 2:** Primers used for quantitative real-time PCR.

Genes	Accession No.	Sequence
*β-actin*	NM_205518.1	F: GTGATGGACTCTGGTGATGGTGTT R: TCTCGGCTGTGGTGGTGAAG
*Nrf2*	NM_205117.1	F: TGTGTGTGATTCAACCCGACT R: TTAATGGAAGCCGCACCACT
*Keap1*	NM_012289.4	F: ACTTCGCTGAGGTCTCCAAG R: CAGTCGTACTGCACCCAGTT
*CAT1*	NM_001031215.2	F: CTATCCTTCCTGGTCTTTCTACAT R: TCATACGCCATCTGTTCTACCT
*Maf F*	NM_204757.2	F: CGACGACGGACGCTGAAGAA R: GTACTTGCCACGGAGAGTGTCAA
*Maf K*	NM_204756.2	F: GCAGCAAGAGGTGGAGAAGC R: ACGGCACGGAACTGGATGA
*Maf G*	NM_001079489.1	F: ACGCTGAAGAACCGAGGCTAC R: GTTCTGGCGAAGTTCTGGAGTG
*GSTT1*	NM_205365.1	F: CATGCTAACATCCGGGCTAA R: AAATTGCTTCAGGGAAGTGG
*GSTA3*	NM_001001777.1	F: GCGGCTGCTGGAGTTGAGTT R: GTAGTTGAGGATGGCTCTGGTCTG
*GSTO1*	NM_001277375.1	F: GGGCTGGTTCCTGTTCTG R: TCTTCTGTAAGGCTCGCTCAT
*SOD1*	NM_205064.1	F: AGGGAGGAGTGGCAGAAGT R: GCTAAACGAGGTCCAGCAT
*SOD2*	NM_204211.1	F: TCCTGACCTGCCTTACGACTATGG R: GCGACACCTGAGCTGTAACATCAC
*NQO1*	NM_001277619.1	F: ACCATCTCTGACCTCTACGCCATA R: GCCGCTTCAATCTTCTTCTGCTC
*HO-1*	NM_205344.2	F: AGTGAGAGGACAAGCAGGATG R: CGACTGTGGTGGCGATGAA
*GCLC*	XM_046915268.1	F: AGGCTATGTGTCCGATATTGATTG R: TGGTTGTTCTTCAGTGGCTCTA
*GCLM*	NM_001007953.2	F: GCTCAGTTAGATTCGGTCATTATTG R: AAGGTCAGAGGTGCCTATGG
*GP* *X* *1*	NM_001277853.2	F: CAAAGTGCTGCTGGTGGTCAAC R: TTGGTGGCGTTCTCCTGGTG
*GP* *X3*	NM_001163232.2	F: TGGCAGAGGAGTTCGGCAAC R: CGTTCTTGACAGTGGCGATGTT
*IκBα*	GR480730.1	F: GTGAGCTTGGTCTGTCGTGT R: TTCATAACTCAGGCCCGCTG
*NF-κB p65*	NM_001396038.1	F: TGAAGAAACGGGAACTGGAAG R: GGCACGGTTGTCATAGATGG
*TNF-* *α*	NM_204267.2	F: GGACAGCCTATGCCAACAAG R: ACCACACGACAGCCAAGT
*I* *FN* *-* *γ*	NM_205427.1	F: GCAACCTTCACCTCACCATC R: CGCTGTAATCGTTGTCTTGGA
*IL-1β*	NM_204524.2	F: GAAGAAGCCTCGCCTGGAT R: TCCGCAGCAGTTTGGTCAT
*IL-2*	AJ224516.1	F: GCTAACTAACCTGCTGTCCATT R: CCGTAGGGCTTACAGAAAGGA
*IL-4*	NM_001007079.2	F: TGACATCCAGGGAGAGGTTTC R: GCAGGTTCTTGTGGCAGTG
*IL-6*	NM_204628.2	F: CCTCCTCGCCAATCTGAAGT R: GCACTGAAACTCCTGGTCTTT
*IL-10*	NM_001004414.4	F: TGTCACCGCTTCTTCACCT R: TCACTTCCTCCTCCTCATCAG
*TFR1*	NM_205256.2	F: AGTTATCGTGGACGAATCGAGC R: ATGACAGGCGGTCCTTGAAT
*Fpn1*	NM_001012913.2	F: CCACAGCGATCACAATTCAGAGG R: CGACATCAGGTTCCAGCCAGAA
*ACSL4*	XM_046917349.1	F: CCGGCAACGTTATCTCCTCC R: GCCTTCTCGCTGTCCTGTAG
*SLC7A11*	XM_426289.7	F: CTGTCGTGACGGTGCCTAA R: CCAATGATAGTGCCAATGATGATG
*GPX4*	NM_204220.3	F: GAATGTGCGCTCAGGCG R: R: ACCGCGGTCTTTCCTCATTT
*PTGS2*	NM_001167718.2	F: TGGTGAGACTCTGGAGAGGCAACT R: GCCAAACACCTCCTGCCCAACA
*Bcl-2*	NM 205339.3	F: GATGACCGAGTACCTGAACC R: CAGGAGAAATCGAACAAAGGC
*Bax*	XM 040693909.1	F: ACTCTGCTGCTGCTCTCCTCTC R: ATCCACGCAGTGCCAGATGTAATC
*Caspase-3*	NM 204725.2	F: CCACCGAGATACCGGACTGT R: AACTGCTTCGCTTGCTGTGA
*Caspase-9*	XM 040689238.1	F: GTGTACCAGCTGCGAGCAGACC R: GCTTTGAGGTTCCGCAGGGTC

**Table 3:** Effects of dietary phlorotannin on hepatic function parameters in the serum of heat-stressed broilers at 42 days old (n = 6).

Items ^1^	Group ^2^			SEM ^3^	*p*-Value			
	TN	HS	HS + PT		ANOVA	TN vs. HS	HS vs. HS + PT	TN vs. HS + PT
TP, g/L	22.11	21.31	23.34	0.79	0.23	0.490	0.098	0.294
ALB, g/L	13.19 ^a^	10.40 ^b^	11.55 ^ab^	0.79	0.087	0.031	0.325	0.173
AST, U/L	28.27	30.30	29.12	1.71	0.71	0.423	0.640	0.732
ALT, U/L	2.26	2.41	2.27	0.18	0.82	0.580	0.600	0.976

^1^ TP: Total protein; ALB: Albumin; AST: Aspartate aminotransferase; ALT: Alanine aminotransferase. ^2^ TN: thermoneutral group, the broilers reared at 24 ± 1 °C throughout the experimental period, fed with basic diet; HS: heat stress group, the broilers reared at 33 ± 1 °C for 8 h/day (9:00 am to 17:00 pm), fed with basal diet; HS + PT: HS + phlorotannin group, the broilers reared at 33 ± 1 °C for 8 h/day (9:00 am to 17:00 pm), fed with 600 mg/kg phlorotannin in the basal diet. ^3^ SEM: the standard error of the mean. ^a,b^ Means in the same row with different letters are significantly different at *p* < 0.05.

**Table 4:** Effects of dietary phlorotannin on the hepatic antioxidant performance of heat-stressed broilers at 42 days old (n = 6).

Items ^1^	Group ^2^			SEM ^3^	*p*-Value			
	TN	HS	HS+PT		ANOVA	TN vs. HS	HS vs. HS + PT	TN vs. HS + PT
T-AOC, mmol/mg protein	0.76	0.70	0.69	0.03	0.196	0.147	0.821	0.101
CAT, U/mg protein	284.25 ^a^	237.84 ^b^	246.45 ^b^	4.55	<0.001	<0.001	0.210	<0.001
T-SOD, U/mg protein	80.71 ^a^	72.87 ^b^	75.54 ^ab^	3.30	0.096	0.037	0.432	0.144
MDA, mmol/mg protein	2.95 ^b^	5.81 ^a^	6.66 ^a^	0.79	0.194	0.029	0.467	0.008
GST, U/mg protein	173.78 ^a^	119.03 ^c^	144.13 ^b^	6.54	0.001	<0.001	0.022	0.009
GSH-Px, U/mg protein	114.04 ^a^	70.36 ^c^	92.31 ^b^	6.15	0.002	0.001	0.030	0.032

^1^ T-AOC: total antioxidant capacity; CAT: catalase; T-SOD, total superoxide dismutase; MDA, malondialdehyde; GST, glutathione S-transferase; GSH-Px, glutathione peroxidase. ^2^ TN: thermoneutral group, the broilers reared at 24 ± 1 °C throughout the experimental period, fed with basic diet; HS: heat stress group, the broilers reared at 33 ± 1 °C for 8 h/day (9:00 am to 17:00 pm), fed with basic diet; HS + PT: HS + phlorotannin group, the broilers reared at 33 ± 1 °C for 8 h/day (9:00 am to 17:00 pm), fed with 600 mg/kg phlorotannin in the basic diet. ^3^ SEM: the standard error of the mean. ^a,b^ Means in the same row with different letters are significantly different at *p* < 0.05.

## Data Availability

All data are contained within the article.
